# The Non-random Location of Autosomal Genes That Participate in X Inactivation

**DOI:** 10.3389/fcell.2019.00144

**Published:** 2019-08-06

**Authors:** Barbara R. Migeon

**Affiliations:** Departments of Genetic Medicine and Pediatrics, The Johns Hopkins University, Baltimore, MD, United States

**Keywords:** single active X, intra-chromosomal interaction, inter-chromosomal interaction, X-chromosome dosage compensation, autosomes in X inactivation, evolutionary conservation, clustered gene interactions

## Abstract

Mammals compensate for sex differences in the number of X chromosomes by inactivating all but one X chromosome. Although they differ in the details of X inactivation, all mammals use long non-coding RNAs in the silencing process. By transcribing XIST RNA, the human inactive X chromosome has a prime role in X-dosage compensation. Yet, the autosomes also play an important role in the process. Multiple genes on human chromosome 1 interact with XIST RNA to silence the future inactive Xs. Also, it is likely that multiple genes on human chromosome 19 prevent the silencing of the *single* active X – a highly dosage sensitive process. Previous studies of the organization of chromosomes in the nucleus and their genomic interactions indicate that most contacts are intra-chromosomal. Co-ordinate transcription and dosage regulation can be achieved by clustering of genes and mingling of interacting chromosomes in 3D space. Unlike the genes on chromosome 1, those within the critical eight MB region of chromosome 19, have remained together in all mammals assayed, except rodents, indicating that their proximity in non-rodent mammals is evolutionarily conserved. I propose that the autosomal genes that play key roles in the process of X inactivation are non-randomly distributed in the genome and that this arrangement facilitates their coordinate regulation.

When female mammals compensate for sex differences in the dosage of X linked genes by inactivating X chromosomes, the X chromosome(s) that is silenced has a major role in the process. In all mammals, a non-coding RNA, encoded by the X, is essential to its being inactivated by epigenetic factors (Grant et al., 2012). Clearly, the bi-directional spread of Xist RNA from its locus in the middle of the X chromosome initiates the inactivation process in eutherian mammals (Brockdorff et al., 1992; Brown et al., 1992). In addition, the other long non coding RNAs, implicated in the process, i.e., the potential *Xist* repressors, rodent-specific *Tsix* (Lee and Lu, 1999), and the primate specific *XACT* (Vallot et al., 2017), are also encoded by the X chromosome. Once coated with enough Xist RNA, the future inactive X moves toward the nuclear lamina, where its chromatin is transformed from euchromatin to heterochromatin (McHugh et al., 2015; Moindrot and Brockdorff, 2016).

The silencing of the future inactive X, or Xs, is attributable to a Rube-Goldberg type of mechanism that not only brings it close to the nuclear periphery (where inactive chromatin tends to reside), but also attracts the epigenetic factors that silence it. Ultimately, the binding of Xist RNA results in expulsion of factors from the inactive X that make chromatin accessible for transcription (Jegu et al., 2019). The few active (escape) genes on that X chromosome manage to find their way out of the heterochromatic mass of inactive chromatin towards the center of the nucleus, where transcription occurs (Fraser and Bickmore, 2007). Yet, Xist RNA cannot do this alone, as autosomal gene products are essential to complete the silencing process (McHugh et al., 2015; Moindrot and Brockdorff, 2016; Patil et al., 2016).

In pursuit of autosomal genes that cooperate with the X chromosome, Percec et al. (2003) used ENU chemical mutagenesis to screen for autosomal mutations involved in the initiation of X inactivation in mice. They identified regions of mouse chromosomes 5, 10, and 15, which seemed to affect the choice of the mouse inactive X. More recent studies in mice have elucidated the essential autosomal products that interact with Xist RNA to silence the chromosome (McHugh et al., 2015; Chen et al., 2016; Moindrot and Brockdorff, 2016; Sunwoo et al., 2017) (Table 1). These include the lamin B receptor (*Lbr)*, the satellite attachment factor A (*Saf-A*) and *Sharp* (*Smrt* and Hdac Associated Repressor Protein, also called *Spen*). *SPEN*, *LBR*, and *SAFA* map to human chromosome 1; *Lbr* and *Safa* also map to mouse chromosome 1, whereas *Sharp* is on mouse chromosome 4 (orthologous to human chromosome 1). Other genes that have been implicated in the silencing process are *RBM 15* and *SETDB1*, on human chromosome 1, and mouse chromosome 3 – also orthologous to human chromosome 1. Therefore, the genes on human chromosome 1 that play a role in silencing the future inactive X also map to mouse chromosome 1 or its orthologs (Table 1 and Figure 1A). Conceivably, genes that were on three different chromosomes in mice have evolved to be on a single human chromosome to facilitate their interaction in silencing the X.

**TABLE 1:** Location of mouse and human genes that silence the inactive X.

**Human GENE**	**Human CHROMOSOME**	**5′ location of Human Gene (*GRch38*)**	**Mouse GENE**	**5′ location of Mouse Gene (*GRCm38*)**	**Citation for Mouse Genes**
SPEN	***1p36.21***	***1:15,847,863*^∗^**	Sharp^∗∗^ (Spen)	***4:141,467,890***	McHugh et al., 2015; Moindrot and Brockdorff, 2016
RBM15	***1p13.3***	***1:110,338,928***	Rbm15	***3:107,325,421***	*McHugh- MoindrotPatil* (Patil et al., 2016)
LBR	***1q42.12***	***1:225,401,501***	Lbr	***1:181,815,315***	*McHugh Chen* (Chen et al., 2016)
HNRNPC	14q11.2	14:21,209,135	Hnrnpc	14:52,073,380	*McHugh*
RALYL	8q21.2	8:84,182,764	Raly Ralyl	***3:13,471,655*** 2:154,791,096	*McHugh*
HNRNPM	19p13.2	19:8,444,574	Hnrnpm	17:33646233	*McHugh*
HDAC3	5q13.3	5:141,620,875	Hdac3	18:37936971	*McHugh*
HNRNPU (SAFA)	***1q44***	***1:244,850,299***	Hnrnpu or Safa	***1:178321108***	*McHugh*
CELF1	11p11.2	11:47,465,932	Celf1	2: 90940387	*Moindrot*
PTBP1	19p13.3	19:797,391	Ptbp1	10:79854432	*McHugh*
Not found			Myef2	2:125,084,628	*Moindrot*
NCOR1	17p12-p11	17:16,030,093	NCoR-Hdac3 complex	11:62316426	*Moindrot*
CIZ1	9q34.11	9:128,166,064	Ciz1	2: 32363005	*Moindrot Sunwoo* (Sunwoo et al., 2017)
SETDB1	***1q21.3***	***1:150,926,245***	Setdb1	***3:95323525***	*Moindrot*
WTAP	6q25.3	6:159,726,695	Wtap	17: 12966799	*Moindrot*
HDAC1	***1p35.2-p35.1***	***1:32,292,102***	Hdac1	***4:129,516,104***	*This paper*

*^∗^Bold italics: Human chromosome 1 or mouse orthologs of human chromosome 1. ^∗∗^SPEN(SMART/HDAC1 associated repressor protein = SHARP.*

**FIGURE 1:**
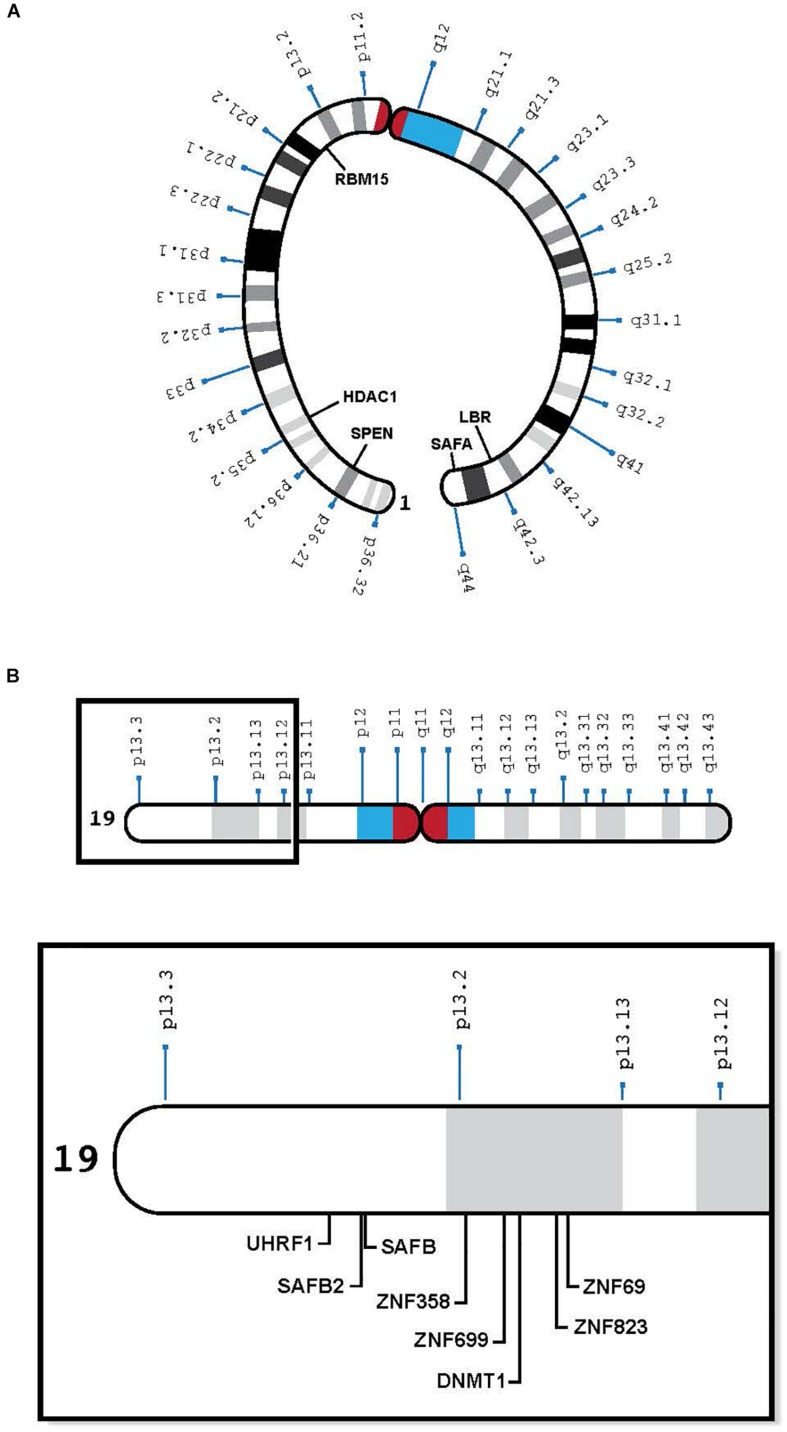
**(A)** Human chromosome 1 with relevant genes, bent to show telomeres in proximity: SPEN 1p36.2, HDAC1 1p35.2, RBM15 1p13.3, LBR 1q42.12, and SAFA 1q44 (see Table 1). **(B)** Human chromosome 19 insert with relevant genes, showing proximity of genes in 19p13.1-13.2: UHRF1, SAFB2, SAFB, ZNF 358, ZNF 699, DNMT1, ZNF 823, and ZNF 69 (see Table 2).

In prokaryotes, interactions between genes with a common function are facilitated because such genes are contiguous in the genome, organized into operons, with a common promoter (Jacob, 2011). On the other hand, most eukaryotic genes that interact with each other, do not share promotors, and are less well clustered (Dekker and Misteli, 2015). Yet, it has become apparent that the spatial arrangement of genes in the mammalian nucleus is non-random; chromosome folding and intermingling enable the proximity of genes that reside on the same chromosome, by looping, and even on different chromosomes, by chromosome clustering. The likely advantage of interactions between genes is coordination of their expression – perhaps in the same transcription factory, thought to occur in a discrete nuclear region (Rieder et al., 2014).

Based on HI-C studies of the human genome (Lieberman-Aiden et al., 2009), Thevenin et al. (2014) showed that a significant number of functional groups (pairs of interacting proteins, genes with common functions and those in interactive pathways) are either clustered within the same chromosome or dispersed over a relatively few chromosomes. Those on different chromosomes tend to co-localize in space. These investigators found that, genes, which function together, tend to reside on fewer chromosomes than expected by chance. On the same chromosome, they are closer to each other than randomly chosen genes; on different chromosomes, they tend to be closer to each other in 3D space (Thevenin et al., 2014). Among the best documented inter-chromosomal interactions are those between the mouse X chromosomal gene, *Xist*, and the autosomal epigenetic factors mentioned above, that help silence the X chromosome from which the up-regulated *Xist* locus is being transcribed (Dekker and Misteli, 2015).

When extending her observations in mice to other mammals, Lyon suggested there was only a single *active* X, no matter the number of X’s in a cell (Lyon, 1962); however, the literature has persisted in labeling the mammalian process of X dosage compensation, X inactivation, which focuses us on the process of silencing the inactive X. Therefore, the salient question has been, “How does one *choose* the X chromosome that becomes *inactive*?” Because Xist RNA is able to silence any chromosome into which it is inserted (Jiang et al., 2013; Migeon et al., 1999), it is surprising that few ask the pertinent question, “What protects the single active X from silencing by its own *Xist* locus?” (Migeon, 2017; Migeon et al., 2017).

Further, it has not been easy to show how the mouse *inactive* X is chosen. Earlier studies suggested that an infrequent physical association (kissing) between the *Xist* loci of the two X chromosomes in mouse embryos determined the choice of inactive X (Xu et al., 2006; Augui et al., 2007), but more recent studies indicate that neither the expression of *Xist* nor *Tsix*, its antisense RNA, is affected by the interaction (Cheng et al., 2019; Pollex and Heard, 2019).

In addition, Inoue et al. (2018) and Harris et al. (2019) recently showed that in mice, the choice of *active* X is determined prenatally. Having been imprinted during oocyte differentiation [as predicted by Lyon and Rastan (1984)], the active X is always *maternal* in trophectoderm – the first tissue to undergo dosage compensation in the mouse embryo. Because X inactivation in the placenta occurs relatively early in mice, it is likely that the paternal X hasn’t had time to erase the inactivation imprint imposed during the early stages of spermatogenesis (Migeon, 2016). It remains to be seen if the rodent specific Tsix RNA, which is transcribed only from the maternal X in trophectoderm, protects the active X, regardless of its parental origin, from silencing by *Xist* in other mouse embryonic tissues.

With respect to human cells, we have learned that (1) human oocytes do not express *PRC2* (which imprints the mouse oocyte) (Harris et al., 2019), (2) the human maternal X is not imprinted (Migeon, 2016), and (3) human *TSIX* is ineffective, having been truncated during human evolution (Migeon et al., 2001). Therefore, another means of repressing the *XIST* locus on the future *active* human X is needed to protect it from being silenced. Recent studies suggest that to prevent its heterochromatization by *XIST*, the future human *active* X needs to interact with human chromosome 19 (Migeon et al., 2017). They reveal a previously unsuspected eight MB region on the short arm of human chromosome 19 (19p13.3-13.2), which contains at least one dosage sensitive gene that is likely to play a role in silencing the *XIST* locus on *one* X chromosome in each cell (Migeon, 2017; Migeon et al., 2017) (Table 2). Candidate genes include satellite attachment factors *SAFB* and *SAFB2*, a cluster of zinc finger proteins that surround *DNMT1* and its co-factor *UHRF1*, among many others. Although most of the zinc finger proteins clustered in the relevant region of human chromosome 19 arose after the split between rodents and humans, the other genes in this region can be found on mouse chromosomes 8, 9, and 17 – orthologous to human chromosome 19 (Table 2 and Figure 1B). Again, perhaps human 19 evolved to facilitate the interaction of genes that protect the future active X.

**TABLE 2:** Location of mouse and human genes that may maintain the active X.

**Human GENE**	**Human CHROMOSOME**	**5’ location of Human Gene *(GRCh38)***	**Mouse GENE**	**5’ location of Mouse Gene *(GRCm38)***	**Citation**
UHRF1	19p13.3	***19:4,903,079***	Uhrf1	***17:56, 303,367***	Migeon et al., 2017
SAFB	19p13.3	***19:5,623,034***	Safb	***17:56, 584,830***	
SAFB2	19p13.3	***19: 5,586,992***	Safb2	***17:56, 560,965***	
DNMT1	19p13.2	***19:10,133,343***	Dnmt1	***9:20,907,209***	
HNRNPM	19p13.2	***19:8,444,574***	Hnrnpm	***17:33, 646,233***	
MBD3	19p13.3	***19:1,576,670***	Mbd3	***10:80,392,539***	
MBD3L-5L	19p13.2	***19:8,842,392***	Mbd3l	***9:18,478, 359***	
PRMT4 or CARM1	19p13.2	***19:10,871,576***	Carm1	***9:21,546,894***	
ZNF358	19p13.2	***19:7,580,178***	Zfp358	***8:3,493,138***	
ZNF699	19p13.2	***19:9,291,139***	*Not found*		
ZNF627	19p13.2	***19:11,575,254***	Znf 867	11:59,461,197	
ZNF823	19p13.2	***19:11,832,080***	*Not found*		
ZNF69	19p13.2	***19:11,887,772***	*Not found*		
ZNF44	19p13.2	***19:12,224,685***	*Not found*		
ZNF443	19p13.2	***19:12,540,521***	Znf 709	***8:71,882,068***	

*Bold italics: Human chromosome 19 or mouse orthologs of human chromosome 19.*

In the genomics era, many human geneticists tend not to specify which particular autosome encodes genes of interest; therefore, I was surprised to see that many of the proteins that interact with *XIST* to silence the X are encoded by human chromosome 1 (Migeon et al., 2017) (Table 1 and Figure 1A), and in the mouse, by the three orthologs of chromosome 1 (chromosomes 1, 3, and 4) (Table 1). In mice, these genes are bound to *Xist* at the same developmental stage (McHugh et al., 2015). To my knowledge, no one has examined the *Xist*-autosomal interactions by RNA FISH to determine if there is clustering of the three murine chromosome 1 orthologs. The positions of these genes on human chromosome 1 is of interest as some of the genes are present on opposing ends of the chromosome, which would require a large fold in the chromosome to facilitate any interaction (Figure 1A). Such intermingling and folding are frequently observed in the 3D nuclear space (Lieberman-Aiden et al., 2009).

Table 3 presents conservation data obtained from the UCSC Genome Browser; it shows that of four relevant genes on chromosome 1 that aid *Xist* in silencing the inactive X, only SAFA and LBR have been on the chromosome since we evolved from marsupials. SPEN and RBM15 although on the same chromosome as SAFA and LBR in primates, are on other chromosomes in marmosets and non-primate mammals. In contrast, except in rodents (rat, mouse, and rabbit), the region on chromosome 19 that protects the active X is preserved in primates such as gorilla, orangutang, and marmoset, and other mammals such as cat, dog, pig, horse, cow, and opposum (Table 4). The exceptional genes that have left the group include the long noncoding RNA, TINCR, and the MD3L3-5, methyl CPG binding domain proteins, which are on chromosome 19 in primates and in marmoset but are not found in all mammals. The conserved cluster in pig, horse and cow is in the reverse orientation (Table 4). These differences interrupt what would otherwise be an exceptionally long synteny block, but the preservation of so many genes in this region, in spite of multiple evolutionary structural alterations, suggests that the local landscape may be important to function. That the chromosome 19 genes in rodents are not conserved as a group argues that their process of ensuring that one X will remain active differs from that of other mammals (Shevchenko et al., 2019), perhaps because only rodents have *Tsix* to protect the active X from silencing by *Xist.*

**TABLE 3:** Conservation of some candidate genes, and not others in various mammals.

**MAMMAL**	**GENE**	**CHROM**	**5′ LOCATION (nucleotides)**	**GENE**	**CHROM**	**5′ LOCATION (nucleotides)**
HUMAN	DNMT1	**19^∗^**	10,133,346	SPEN	**1**	15,847,864
	UHRF1	**19**	4,910,367	LBR	**1**	225,401,503
	SAFB	**19**	5,623,099	SAFA	**1**	244,850,297
	SAFB2	**19**	5,586,999	RBM15	**1**	110,286,375
GORILLA	DNMT1	**19**	9,911,947	SPEN	**1**	15, 818,157
	UHRF1	**19**	4,549,324	LBR	**1**	205,129,423
	SAFB	**19**	5,391,167	SAFA	**1**	224,804,897
	SAFB2	**19**	5,343,115	RBM15	**1**	111,770,116
ORANGUTAN	DNMT1	**19**	10,128,395	SPEN	**1**	212,361,620
	UHRF1	**19**	4,819,523	LBR	**1**	24,182,913
	SAFB	**19**	5,532,720	SAFA	**1**	4,279,561
	SAFB2	**19**	5,496,867	RBM15	**1**	116,356,665
MARMOSET	DNMT1	**22**	9,536,311	SPEN	7	50,174,237
	UHRF1	**22**	4,640,990	LBR	**19**	18,374,272
	SAFB	**22**	5,347,272	SAFA	**19**	35,988,006
	SAFB2	**22**	5,310,815	RBM15	7	146,230,306
Pig	DNMT1	**2**	68,982,341	SPEN	6	75,015,891
	UHRF1	**2**	73,898,195	LBR	**10**	13,389,915
	SAFB	**2**	73,300,630	SAFA	**10**	17,485,493
	SAFB2	**2**	73,334,753	RBM15	4	109,778,998
COW	DNMT1	**7**	15,914,205	SPEN	**16**	52,882,374
	UHRF1	**7**	20,436,673	LBR	**16**	29,148,981
	SAFB	**7**	19,846,024	SAFA	**16**	33,162,888
	SAFB2	**7**	19,908,323	RBM15	3	33,196,547
SHEEP	DNMT1	**5**	12,315,683	SPEN	**12**	49,635,296
	UHRF1	**5**	16,747,203	LBR	**12**	26,512,015
	SAFB	**5**	16,167,299	SAFA	**12**	30,479,650
	SAFB2	**5**	16,230,105	RBM15	1	86,670,575
HORSE	DNMT1	**7**	49,751,153	SPEN	2	37,048,480
	UHRF1	**7**	3,014,835	LBR	**30**	8,017,554
	SAFB	**7**	3,409,307	SAFA	**30**	0,184,656
	SAFB2	**7**	3,388,372	RBM15	5	57,896,671
DOG	DNMT1	**20**	50,880,023	SPEN	2	81,683,829
	UHRF1	**20**	54,858,675	LBR	**7**	39,291,511
	SAFB	**20**	54,381,519	SAFA	**7**	35,833,232
	SAFB2	**20**	54,381,353	RBM15	6	41,645,939
CAT	DNMT1	**A2**	7,689,975	SPEN	1	11,528,828
	UHRF1	**A2**	3,678,067	LBR	**F1**	1,574,749
	SAFB	**A2**	4,176,193	SAFA	**F1**	5,103,486
	SAFB2	**A2**	4,143,427	RBM15	1	94,297,141
OPPOSUM	DNMT1	**3**	431,238,772	SPEN	4	375,579,105
	UHRF1	**3**	441,797,772	LBR	**2**	137,055,167
	SAFB	**3**	443,046,263	SAFA	**2**	142,860,792
	SAFB2	**3**	443,045,746	RBM15	**2**	479,908,213

*^∗^Chromosome numbers in bold indicate conservation.*

**TABLE 4:** Site of genes on human chromosome 19 in other mammals.

**MAMMAL**	**GENE**	**CHROMOSOME**	**SITE 5’ (nucleotide)**
HUMAN	SIRT6	19	4,174,109
	PLIN3	19	4,852,208
	UHRF1	19	4,910,367
	KDM4B	19	4,969,121
	TINCR	19	5,560,774
	RFX2	19	5,993,164
	VAV1	19	6,772,726
	MBD3L4	19	7,037,748
	INSR	19	7,112,226
	ZNF358	19	7,516,118
	MAP2K7	19	7,903,891
	FBN3	19	8,130,286
	HNRNPM	19	8,269,278
	ZNF558	19	8,806,170
	OLFM2	19	9,853,718
	DNMT1	19	10,133,346
	DNM2	19	10,828,755
	CARM1	19	10,871,513
ORANGUTAN	SIRT6	19	4,083,376
	PLIN3	19	4,752,733
	UHRF1	19	4,819,523
	KDM4B	19	4,940,648
	TINCR	19	5,468,562
	RFX2	19	5,907,338
	VAV1	19	6,738,253
	MBD3L4	19	7,005,357
	INSR	19	7,065,165
	ZNF358	19	7,328,128
	MAP2K7	19	7,862,957
	FBN3	19	8,037,199
	HNRNPM	19	8,412,645
	ZNF558	19	8,801,446
	OLFM2	19	9,841,684
	DNMT1	19	10,128,395
	DNM2	19	10,719,521
	CARM1	19	10,872,517
MARMOSET	SIRT6	22	3,843,381
	PLIN3	22	4,576,676
	UHRF1	22	4,640,990
	KDM4B	22	4,753,547
	TINCR	22	5,280,800
	RFX2	22	5,714,269
	VAV1	22	6,482,055
	MBD3L4	22	6,745,638
	INSR	22	6,884,705
	ZNF358	22	7,258,135
	MAP2K7	22	7,564,197
	FBN3	22	7,702,224
	HNRNPM	22	8,116,508
	ZNF558	22	8,418,995
	OLFM2	22	9,242,165
	DMNT1	22	9,536,311
	DNM2	22	10,141,800
	CARM1	22	10,298,967
PIG	SIRT6	2	74,568,548
	PLIN3	2	73,970,200
	UHRF1	2	73,898,195
	KDM4B	2	73,747,610
	RFX2	2	72,949,979
	*TINCR*	*not found*	
	VAV1	2	72,327,498
	MBD3L4	2	72,012,690
	INSR	2	71,797,542
	ZNF358		71,615,476
	MAP2K7	2	71,298,318
	FBN3	2	71,104,118
	HNRNPM	2	70,813,749
	ZNF558	2	70,582,106
	OLFM2	2	68,734,136
	DMNT1	2	68,982,341
	DNM2	2	69,474,069
	CARM1	2	69,602,214
HORSE	SIRT6	7	2,539,099
	PLIN3	7	2,972,664
	UHRF1	7	3,014,835
	KDM4B	7	3,087,218
	RFX2	7	3,649,694
	*TINCR*	*not found*	
	VAV1	7	4,329,609
	*MBD3L4*	*7*	*52,446,746*
	INSR	7	4,882,687
	ZNF358	7	4,701,725
	MAP2K7	7	5,229,948
	FBN3	7	5,361,278
	HNRNPM	7	52,895,099
	*ZNF558*	*7*	*52,539,233*
	OLFM2	7	49,967,570
	DMNT1	7	49,751,153
	DNM2	7	49,316,987
	CARM1	7	49,257,318
COW	SIRT6	7	21,079,141
	PLIN3	7	20,507,000
	UHRF1	7	20,436,673
	KDM4B	7	20,308,693
	RFX2	7	19,126,799
	*TINCR*	*not found*	
	VAV1	7	18,866,379
	MD3L4	7	17,264,390
	INSR	7	17,276,143
	ZNF358	7	17,610,070
	MAP2K7	7	17,891,887
	FBN3	7	18,005,675
	HNRNPM	7	18,289,395
	ZNF558	7	17,220,537
	OLFM2	7	15,550,353
	DNMT1	7	15,914,205
	DNM2	7	16,465,942
	CARM1	7	16,571,428
DOG	SIRT6	20	55,416,563
	PLIN3	20	54,924,119
	UHRF1	20	54,858,675
	KDM4B	20	54,715,308
	RFX2	20	54,013,618
	*TINCR*	*not found*	
	VAV1	20	53,482,255
	MBD3L4	20	53,213,540
	INSR	20	52,017,347
	ZNF358	20	52,314,421
	MAP2K7	20	52,594,536
	FBN3	20	52,723,997
	HNRNPM	20	52,997,963
	ZNF558	20	51,897,297
	OLFM2	20	51,148,154
	DMNT1	20	50,880,023
	DNM2	20	50,399,784
	CARM1	20	50,331,081
CAT	SIRT6	A2	3,162,759
	PLIN3	A2	3,631,793
	UHRF1	A2	3,678,067
	KDM4B	A2	3,765,143
	RFX2	A2	4,427,650
	*TINCR*	*not found*	
	VAV1	A2	5,108,402
	*MBD3L4*	A2	5,395,765
	INSR	A2	6,443,171
	ZNF358	A2	6,267,306
	MAP2K7	A2	6,004,415
	FBN3	A2	5,820,368
	HNRNPM	A2	5,569,560
	ZNF558	A2	6,657,696
	OLFM2	A2	7,484,626
	DMNT1	A2	7,689,975
	DNM2	A2	8,118,334
	CARM1	A2	8,257,736
OPOSSUM	SIRT6	3	440,652,009
	PLIN3	3	441,702,797
	UHRF1	3	441,797,772
	KDM4B	3	441,910.670
	RFX2	3	443,674,276
	*TINCR*	*not found*	
	VAV1	3	444,980,624
	*MBD3L4*	*not found*	
	INSR	3	463,520,164
	ZNF358	*not found*	
	MAP2K7	3	462,757,443
	FBN3	3	461,508,720
	HNRNPM	3	460,359,655
	*ZNF558*	4	*409,014310*
	OLFM2	3	431,554,923
	DMNT1	3	431,238,772
	DNM2	3	430,280,994
	CARM1	3	430,212,862
MOUSE	SIRT6	10	81,621,787
	PLIN3	17	56,277,475
	UHRF1	17	56,304.407
	KDM4B	17	56,326,074
	RFX2	17	56,775,897
	*TINCR*	*not found*	
	VAV1	17	57,279,100
	*MBD3L4*	*not found*	
	INSR	8	3,150,922
	ZNF358	8	3,493,154
	MAP2K7	8	4,238,740
	FBN3	18	58,012,265
	HNRNPM	17	33,646,236
	*ZNF558*	*not found*	
	OLFM2	9	20,672,332
	DNMT1	9	20,907,209
	DNM2	9	21,425,244
	CARM1	9	21,546,894
RAT	SIRT6	7	10,937,622
	PLIN3	9	10,774,869
	UHFR1	9	10,738,211
	KDM4B	9	10,656,035
	RFX2	9	10,216,249
	*TINCR*	9	10,499,290
	VAV1	9	9,617,783
	*MBD3L4*	*8*	*18,226,238*
	INSR	12	1,678,623
	ZNF358	12	2,046,542
	MAP2K7	12	2,546,139
	FBN3	18	53,070,463
	HNRNPM	7	18,516,253
	*ZNF558*	*not found*	
	OLFM2	8	21,684,494
	DNMT1	8	21,922,515
	DNM2	8	22,458,869
	CARM1	8	22,527,213
RABBIT	SIRT6	3	16,044,566
	PLIN3	*not found*	
	UHRF1	1	47,672,908
	KDM4B	1	47,085,460
	RFX2	1	51,045,589
	*TINCR*	*not found*	
	VAV1	13	56,144,807
	*MBD3L4*	*unknown*	
	INSR	un0069	1,077,773
	ZNF358	un0069	914,737
	MAP2K7	un0069	665,019
	FBN3	3 un0069	11,898,428 502,497
	HNRNPM	un0069	252,960
	ZNF558	*not found*	
	OLFM2	un0135	324,580
	DNMT1	un0135	156,550
	DNM2	13	20,368,794
	CARM1	1	51,421,465

Most likely, the relevant genes on the same chromosome are co-regulated. The advantage of genes clustered in interphase is that they can be programmed for simultaneous transcription. To silence *XIST* on the future active X, some genes in the chromosome 19 cluster might be transcribed together, perhaps if they are close enough in 3D space, as a single transcript. The telomeric location of genes on primate chromosome 1 that participate in *XIST* silencing (Figure 1A) suggest that the two ends of the chromosome might physically interact at the time of transcription.

Several important questions remain unanswered: First, how do multiple genes in the inactivation pathway on human chromosome 1 (or in the activation pathway on chromosome 19) coordinately interact with each other? And then, how do autosomal genes encoding protein products, interact with the X chromosome?

Recent studies suggest that the intra-chromosomal gene interactions occur within the same topologically-associating-domain (TAD) (Nora et al., 2012; Galupa and Heard, 2018) and that TADS align with co-coordinately regulated gene clusters, fostering long-range contacts and preventing deleterious interactions between genes in different TADs (Galupa and Heard, 2018) One would like to examine the candidate genes on human chromosomes 1 and 19, at the appropriate time in development, to determine if they are located within the same TAD, or are otherwise coordinately regulated. It is unlikely that the occurrence of multiple silencers of the inactive X on human chromosome 1 and *XIST* repressors on human chromosome 19 is coincidental.

The question of how genes on an autosome interact with the genes on the X chromosome is especially challenging because in the human species either one or several X chromosomes can be silenced within a cell, the number dependent upon the number of X chromosomes in the genome. All but one X chromosome are silenced no matter how many are in the cell, nor the sex of the individual (Grumbach et al., 1963). Therefore, only one X chromosome *resists* silencing no matter the number of X chromosomes in the cell.

Clearly, suppressing the *XIST* locus on the future active X is easier for males than females. We know this because of the specific loss of females who reduplicate the essential chromosome 19 gene(s), presumably because reduplication enables both X’s to be active – a known lethal event in diploid cells. At least five percent more pre-implantation human females are miscarried than are males (Migeon et al., 2017). If males reduplicate the *XIST* repressor, it has little consequence, but females who by chance inactivate both *XIST* loci, die before they implant into the uterus. This suggests that not only when this region of chromosome 19 is duplicated, but even, when the chromosome is normal, the required interaction is a difficult one, as either too little or too much *XIST* repressor would lead to a lethal event (too many active X’s or no active X). The former does not occur as often in males who have only one X chromosome: too much repressor is not lethal, although too little might be.

And there is the question of gene dosage. How in a diploid cell do two autosomes cooperate to make an inhibitor for a single X chromosome? In the case of more than two X chromosomes, how is the right dosage of gene product from chromosome 1 achieved? On one hand Lyon (1971) and more recently Nguyen et al. (2019) suggest that the two autosomes might pair to synthesize a single product. One such product might be a dimeric protein, there is also the possibility of competitive inhibition. Once, a molecule of gene product arrives on one X chromosome then the other(s) are unable to be hit. On the other hand, perhaps, not all attempts to activate or inactivate the chromosome are successful, and so the process is stochastic. That many errors occur while repressing *XIST* on the future active X might explain a significant loss of pre-implantation females, even in absence of gene reduplication.

To answer these questions one needs to identify genome interactions during the pre-implantation development of the human embryo, at the time of X inactivation. One can use chromosome capture such as Hi-C, 3D RNA-FISH (Shiura and Abe, 2019) (to see if nascent transcripts are transcribed together). Single-cell RNA-Seq as has been recently described in the mouse (Cheng et al., 2019), examining the candidate genes. The best human model would be the beginning of cleavage to embryonic day 10. The inability to study available human embryos is a decided disadvantage for American investigators, but I hope that my colleagues in other countries will carry out such studies. For the human X: 19 interaction, embryonic day 4–7 would probably be appropriate, whereas human embryonic day 6–9 should capture the chromosome 1: X interaction.

## Author Contributions

BM conceived the study, obtained the data, and wrote the manuscript.

## Conflict of Interest Statement

The author declares that the research was conducted in the absence of any commercial or financial relationships that could be construed as a potential conflict of interest.
